# Heterogeneity, Self-Renewal, and Differentiation of Hematopoietic Stem Cells

**DOI:** 10.1155/2012/580165

**Published:** 2012-05-21

**Authors:** Roland Jurecic, Linheng Li, R. Keith Humphries

**Affiliations:** ^1^Department of Microbiology and Immunology, Sylvester Comprehensive Cancer Center, Miller School of Medicine, University of Miami, Miami, FL 33136, USA; ^2^Department of Pathology and Laboratory Medicine, Stowers Institute for Medical Research, University of Kansas School of Medicine, Kansas City, MO 64110, USA; ^3^Terry Fox Laboratory, British Columbia Cancer Agency and Department of Medicine, University of British Columbia, Vancouver, BC, Canada V5Z 1L3

Better understanding of how hematopoietic stem cells (HSCs) balance self-renewal and differentiation is vital for their application in treatment of various diseases and regenerative medicine. During the last decade tremendous advances have been made in identifying a complex network of cellular and molecular factors that influence self-renewal and differentiation of HSCs and characterizing the cellular and molecular components of niches which harbor HSCs. Improving our understanding of the cellular and molecular mechanisms that regulate self-renewal and differentiation of HSCs will have a profound impact on experimental and clinical HSCs research and clinical application of HSCs. Thus, *Stem Cells International *set out to publish a special issue devoted to the topic of *heterogeneity, self-renewal, and differentiation of hematopoietic stem cells*.

The ongoing research is continuously increasing our knowledge about HSC niches in the bone marrow and in particular about cell types constituting niches and a plethora of molecules and signals that make up the synapse between HSCs and niche cells. A series of three review articles in this issue discuss the role of hematopoietic niches in regulating behavior and function of hematopoietic stem cells and the role of niches in development of leukemias. 

K. S. Tieu et al. present a review of quantitative approaches to understanding stem cell niche signaling in the hematopoietic system, as well as in other tissues under conditions of homeostasis and carcinogenesis. They explain the benefits of mathematical models in advancing our understanding of the mechanisms that regulate stem cell fate and how this regulation changes in cancer development. K. S. Tieu and colleagues are also highlighting the synergistic relationship between mathematical predictions and experimental validation and address the potential for mathematical models to predict and optimize therapies targeting the stem cell niche.

In their review “*Osteoblastic and vascular endothelial niches, their control on normal hematopoietic stem cells, and their consequences on the development of leukemia*” B. S. Guerrouahen and colleagues provide some common cellular and molecular principles applying to the osteoblastic and vascular hematopoietic niches and discuss altered microenvironment signaling leading to myeloid lineage disease. They also review the emerging evidence for the role of microenvironment in supporting the leukemia-initiating cells (LICs) and the influence of the microenvironment on chemotherapy resistance.

The two predominant niches in the bone marrow, the endosteal and vascular niches, are thought to regulate the self-renewal and differentiation of distinct HSC populations, and also dictate HSC behavior with respect to homeostatic requirements and exogenous stresses. In their review article “*The Haematopoietic stem cell niche: new insights into the mechanisms regulating haematopoietic stem cell behaviour*” A. J. Lilly and colleagues discuss recent research into the cellular and molecular components of endosteal and vascular niches. Taking into account the crosstalk and overlap between cell types and signaling in these two niches, J. Lilly et al. propose that endosteal and vascular niches should be viewed as subcompartments of a single HSC niche. The review by H. C. O'Neill et al. from Australia focuses on the spleen microenvironment as a site of development of novel dendritic-like cells which are phenotypically and functionally distinct from other described antigen-presenting cells. The discovery that the lineage origin and the progenitors for this new type of tissue-specific antigen-presenting cells differ from that of other known dendritic and myeloid cell types suggests that spleen represents a distinct microenvironment for development of a novel myeloid cell type arising from HSCs or progenitors endogenous to spleen. This paper also highlights the need to explore in more detail the contribution of the spleen and its microenvironment to steady-state hematopoiesis.

**β**-Thalassemia is characterized by reduction or absence of **β**-globin production, resulting in anemia. Current therapies include blood transfusion combined with iron chelation, BM transplantation which is restricted by the matched donor limitation, and gene therapy with **β**-globin lentiviral vectors. The review by E. Drakopoulou et al. presents the current status of gene therapy for **β**-thalassemia, its success and limitations, and the novel promising strategies available involving the therapeutic role of HSCs. This paper reviews achievements in improving vector safety and efficiency to stably transduce HSCs, while minimizing insertional mutagenesis. The authors also discuss strategies that result in higher numbers of genetically modified HSCs, including manipulation of the ex vivo HSC culture conditions, use of mobilized HSC, and generation of HSCs from patient-specific induced pluripotent stem (iPS) cells. These novel strategies could set the ground for more successful **β**-thalassemia gene therapy clinical trials.

The papers in this special issue highlight both the advancements and challenges in understanding the role of niches in HSC biology and fate and in the use of HSCs in disease treatment.

*Roland Jurecic* *Roland Jurecic* *Linheng Li* *Linheng Li* *R. Keith Humphries* *R. Keith Humphries*

